# A fully humanized IgG-like bispecific antibody for effective dual targeting of CXCR3 and CCR6

**DOI:** 10.1371/journal.pone.0184278

**Published:** 2017-09-05

**Authors:** Remy Robert, Laurent Juglair, Ee X. Lim, Caroline Ang, Carl J. H. Wang, Gregor Ebert, Olan Dolezal, Charles R. Mackay

**Affiliations:** 1 Department of Biochemistry and Molecular Biology, Biomedicine Discovery Institute, Monash University, Clayton, Victoria, Australia; 2 Monash Antibody Technologies Facility (MATF), Monash University, Clayton, Victoria, Australia; 3 Division of Infection and Immunity, The Walter and Eliza Hall Institute of Medical Research, Parkville, Victoria, Australia; 4 Materials Science and Engineering, CSIRO, Parkville, Victoria, Australia; Institut Cochin, FRANCE

## Abstract

Chemokines and their receptors are pivotal for the trafficking of leukocytes during immune responses, and host defense. However, immune cell migration also contributes to a wide variety of autoimmune and chronic inflammatory diseases. Compelling evidence suggests that both CXCR3 and CCR6 chemokine receptors play crucial roles in the migration of pathological Th1 and Th17 cells during the course of certain inflammatory diseases. The use of two or more receptors by pathogenic cells may explain why targeting of individual receptors has proven disappointing in the clinic. We therefore hypothesized that simultaneous targeting of both CXCR3 and CCR6 with a bispecific antibody (BsAb) might result in decreased chemotaxis and/or specific depletion of pro-inflammatory T cell subsets. In this study, we designed and characterized a fully humanized BsAb. We show that the BsAb binds to both chemokine receptors, as demonstrated by Flow Cytometry and Surface Plasmon Resonance analysis. Furthermore, we demonstrate that the BsAb effectively blocks cell chemotaxis and induces specific antibody-dependent cell-mediated cytotoxicity (ADCC) *in vitro*. Therefore, we propose that dual targeting of CXCR3 and CCR6 with a fully humanized BsAb may display a potent interventional approach for the treatment of inflammatory and autoimmune diseases.

## Introduction

Chemokine receptors control the movement and the positioning of distinct subsets of immune cells [1]. Since the accumulation of leukocytes in certain tissues contributes to a wide variety of inflammatory diseases, selective targeting of certain chemokine receptors represents one promising approach for the development of new anti-inflammatory drugs [2]. Indeed, chemokine-chemokine receptor interactions are a central event for recruitment of immune cells in chronic inflammatory and/or autoimmune diseases. The accumulation of both IFN-γ producing T helper type 1 (Th1) and IL-17-producing Th17 have been strongly implicated in the pathogenesis of Rheumatoid Arthritis (RA), Multiple Sclerosis (MS) and other autoimmune diseases [3–5]. Therefore, the development of anti-inflammatory drugs that are capable of inhibiting the migration or survival of these different T-cell subsets offers potential for selective immunotherapy.

Certain chemokine receptors are preferentially expressed on specific T cell subsets. For example, the CXCR3 receptor is mostly expressed on activated Th1 cells and binds its pro-inflammatory ligands CXCL10 (IP-10), CXCL9 (MIG) and CXCL11 (I-TAC) [6]. Importantly, CXCR3 is highly expressed by T-cells associated with chronic inflammation and/or autoimmune conditions such as RA, Ulcerative Colitis, Lupus or organ transplant rejection and CXCR3 inhibitors have been shown to be beneficial in preclinical disease models of these pathological conditions [7, 8].

On the other hand, CCR6 is mainly expressed by Th17 cells [9, 10]. The expression of CCL20 (MIP3α), the sole ligand for CCR6, is dramatically increased in numerous inflammatory and/or autoimmune conditions and is involved in the recruitment of Th17 cells to inflamed sites [11–14]. Various studies using CCR6-deficient mice or CCR6 inhibitors indicate the potential of this receptor as a therapeutic target in autoimmune disease [4, 11, 15]. Importantly, it has been recently shown that pro-inflammatory human Th17 cells are restricted to a subset expressing both CCR6 and CXCR3 (Th17.1) [16]. Another promising therapeutic approach is the specific depletion of pathological lymphocyte subsets, without affecting the function of other leukocytes required for protective immunity. This strategy has been successfully employed in RA and MS models using a monoclonal antibody targeting the lymphotoxin-α receptor, which is expressed by Th1 and Th17 cells [17].

However, therapeutic interventions that target a single chemokine receptor to inhibit immune cell migration have shown to be restricted to a limited number of immune diseases [2]. Moreover, therapeutic approaches that target broad cell migration may be immune-compromising [18]. Importantly, immune diseases often involve several migration pathways of immune cells and multiple chemokine/receptor interactions [2]. To ultimately prevent the recruitment of mixed leukocyte subsets to the inflamed tissue, these diseases require a combination of blocking agents to achieve maximal therapeutic benefit.

In this study, our aim was to develop a humanized bispecific antibody (BsAb) that is capable of binding, simultaneously, CXCR3 expressed by Th1; CCR6 expressed by Th17 or CXCR3 and CCR6 co-expressed by pathogenic Th17.1. Over the last decade, a range of BsAbs formats have emerged [19]. Here, we describe the generation and characterization of a fully humanized, tetravalent BsAb composed of a complete IgG1 with a C-terminal stabilized single-chain Fv (scFv). Because this format retains an intact Fc region, our BsAb offers the advantages of (i) preserving the immunological effector functions of antibody dependent cell cytotoxicity (ADCC), (ii) retaining acceptable pharmacokinetic properties and (iii) an extended serum half-life.

Our BsAb, purified from the supernatant of stably transfected Chinese Hamster Ovary (CHO) cells, demonstrated specific binding to both receptors, CXCR3 and CCR6, as verified by Flow Cytometry and Surface Plasmon Resonance (SPR) analysis. In addition, we were able to show that the our BsAb induces specific ADCC, as well as potent inhibition of immune cell migration.

To our knowledge, this is the first comprehensive description of a bispecific antibody that is capable of simultaneously binding to CXCR3 and CCR6, two highly promising targets for inflammatory and autoimmune disorders.

## Materials and methods

### Ethics statement

All animal procedures were conducted in accordance with the Australian National Health and Medical Research Council (NHMRC) guidelines and were approved by the Monash Animal Research Platform (MARP) Animal Ethics Committee (AEC) (MARP/2016/034). Male C57BL/6 mice, aged 6–10 weeks were purchased from Monash Animal Research Platform (Monash University) and were housed in specific pathogen free conditions. The animals had free access to water and food throughout experiments and were reviewed daily by both the researchers and animal facility staff.

Mice were humanely euthanized with carbon dioxide at the completion of experiments or if mice showed any signs of the following: lethargy, persistent recumbency, hunched posture, rough coat or loss of body condition.

Buffy coats were obtained from blood donations of healthy donors were conducted in accordance with the Declaration of Helsinki, and kindly provided by the Australian Red Cross Blood Service. Their use for this project was approved by Monash University Human Research Ethics Committee (MUHREC) (CF15/3750–2015001629).

### Cell lines and antibodies

Murine pre–B cell lymphoma L1.2 cells [20] were transfected (NucleofectorTM, Lonza Inc.) with a mammalian expression vector pcDNA 3.1 (+) (Invitrogen) containing the human CCR6 or human CXCR3 genes. G418–selected transfectants with highest levels of expression were sorted and used for immunization. Monoclonal antibodies reactive with CCR6 and CXCR3 were generated by injecting C57BL/6 mice (6–10 weeks of age) intraperitoneally (i.p.) with 10^7^ L1.2 human CCR6 or human CXCR3 transfected cells, respectively, fortnightly, five times. The final immunization was injected intravenously (i.v.) 4 days later, the spleen was removed and cells were fused with the SP2/0 cell line (ATCC:1581) as described [21].

### Humanization of the X3 and R6 antibodies by CDR grafting

Total RNA, extracted from the generated X3 and R6 hybridoma cell lines using Trizol (Invitrogen), were used for cDNA synthesis. The V genes were isolated from cDNA by RT–PCR using primers annealing to the mouse light (mIgCk-1: 5’-CTTCCACTTGACATTGATGTCTTTG-3’) and the heavy constant region (mIgG2a-1: 5’-CAGGTCAAGGTCACTGGCTCAGG-3’). The VH and VL genes were sequenced and analyzed by the IMGT databases.

IMGT/V-QUEST [22] and IMGT/Junctions [23] analysis tools were used to identify human germline genes. The sequences from the variable regions of both, the heavy and light chains, were closely aligned with those of murine antibody X3 and R6 as described previously [24]. Framework sequences of these selected human germline genes were used as acceptor sequences for the X3 and R6 CDRs. Importantly, murine residues were retained in the critical ‘‘Vernier” zone. The humanized VH and VL genes were synthesized by GENEART AG (Regensburg, Germany).

### Generation, expression and purification of recombinant scFv

To construct the humanized X3 (hX3) and R6 (hR6) scFv, the VH and VL genes were amplified with primers hX3 VH-FOR (5’-GCCATAATGCTCTTCCTCC-3’); hX3 VH-REV (5’-GCCATTATGCTAGCAGAGGACACGGTCACGGTGG-3’); hX3 VK-FOR (5’-GCCATATACGTCTCACTCCGACATCCAGATGACCCAGTC-3’); hX3 VK-REV (5’-GCCATTAACGTACGCTTGATTTCCACCTTGGTGC-3’); hR6 VH-FOR (5’-GCCATAATGCTCTTCCTCCGAGGTGCAGCTGGTGGAATC-3’); hR6 VH-REV (5’-GCCATTATGCTAGCAGAGGACACGGTGACCAGGG-3’); hR6 VK-FOR (5’-GCCATATACGTCTCACTCCGACATCGTGATGACCCAGTC-3’) and hR6 VK-REV (5’-GCCATTAACGTACGCTTGATTTCCAGCTTGGTGC-3’). The PCR products were assembled and cloned into the pGC expression vector [25].

Transformed E. coli XL-1 Blue cells (Stratagene, La Jolla, CA, USA) were grown at 30°C in 1 L of 2 YT medium containing glucose (0.1%) and ampicillin (100 lg/mL). When OD600 = 1 was reached, isopropyl b-D-thiogalactopyranoside was added to a final concentration of 0.1 mM, and the temperature was reduced to 26°C. After 4 h of protein induction, the cells were pelleted and a periplasmic extract was prepared by sequential extraction with ice-cold 1 x TES buffer (0.2M Tris-HCl, pH 8.0, 0.5 mM EDTA, and 0.5M sucrose) and 0.2 x TES buffer. The periplasm extract was clarified by centrifugation (20,000g; 30 min) and filtered through a0.45-μm membrane (Millipore).

### Three-dimensional structure modeling of the antibody fragments

The molecular model of the mX3; hX3; mR6 and hR6 scFvs were obtained by using the web antibody modeling (WAM) algorithm [26]. Images of the model were generated using PyMOL software (PyMOL version 0.82, http://pymol.sourceforge.net/) (DeLano Scientific LLC).

### Mutagenesis of the antibody fragments

Site-directed mutagenesis of the hX3 scFv was performed using the Quick Change Site-directed Mutagenesis Kit II (Stratagene). Mutations were verified by sequencing the coding region of the antibody fragments on both strands.

### Generation; expression and purification of humanized BsAb

The hCXCR3 x hCCR6 BsAb was designed as a C-terminal heavy chain scFv construct for expression in CHO-DG44 cells (Invitrogen) using our “in house” pJacq expression vector. First, a (Gly_4_S)_3_ linker was inserted by PCR at the C-terminal end of the human IgG1 Fc region and the resulting PCR product was cloned in the pGem-T easy vector. The gene coding for the stabilized hX3 scFv was next amplified and cloned into the pGem-T-easy vector (Promega) containing the hIgG1-inker cassette using BamH1 and Cla1 restriction sites. The hIgG1 Fc-(Gly4S)3-hX3 scFv was then subcloned in pJacq mammalian expression vector. In a final step, hR6 VH and VL genes were cloned into pJacq. The ligation mixtures were transformed into XL-1 E. coli. DNA sequence analysis was used to confirm the correct sequence of the BsAb construct.

### Expression and purification of humanized BsAb

CHO-DG44 cells were transfected with plasmid DNA encoding BsAb using an AMAXA instrument (Lonza). Transformants were selected in Opti-CHO media for their abilities to grow without HAT. After 15 day, the cells that grow without HAT were cloned using ClonePix FL (Genetix). Briefly, the cells were plated in semi-solid medium (Clone Matrix, Genetix) and supplemented with FITC-anti-human IgG antibody (Clone Detect, Genetix) for the capture of the secreted BsAb. Clones were analyzed using parameters of fluorescence intensity, brightness, size, shape and distance between colonies using ClonePix FL software (Genetix). The selected colonies were plated in a 96-well culture plate containing Opti-CHO medium supplemented with 2 mM L-Glutamine. Antibody production was monitored in the supernatant of growing colonies by quantitative Western blot. The best producing clone was next scaled up for BsAb production in 1 L roller bottle. After 10 days, the supernatant was harvested and purified on a ProSep-Va (Millipore) column as previously described [25]. To remove aggregated materials, the BsAb was further purified by gel filtration chromatography through a Superdex^®^ 200 HR 10/30 column (Amersham Pharmacia Biotech).

### Flow cytometry analysis

To assess reactivity of mAbs, BsAbs and scFvs against transfected cells, we used indirect immunofluorescence staining and flow cytometry. Cells were washed once with PBS and resuspended in 100 μl PBS containing 2% (wt/vol) BSA and 0.1% (wt/vol) sodium azide (staining buffer) and the purified recombinant antibody. After 30 min at 4°C, cells were washed twice with staining buffer and resuspended in 50 μl PE-conjugated anti-human IgG (Jackson ImmunoResearch Laboratories), diluted 1:500 in staining buffer for the detection of humanized mAbs and BsAbs. For the detection of scFvs, we used an anti-his tag antibody (QIAGEN). After incubating for 20 min at 4°C, cells were washed twice with staining buffer and analyzed on LSRII flow cytometer (BD Biosciences). 7-AAD staining was used to exclude dead cells. To assess reactivity of mAbs against human lymphocytes, fresh human PBMCs were prepared from buffy coats (obtained from healthy individuals) by centrifugation on a Ficoll gradient [27]. PMBCs were then incubated with anti-CD3-BV650 (clone OKT3, Biolegend) and anti-CD4-Pacific Blue (clone OKT4, Biolegend). The lymphocytes in the CD3+/CD4+ gate were next stained with the hX3 mAb; hR6 mAb or BsAb followed by an anti-human Kappa PE-conjugated antibody (Southern Biotech). In some experiment, hR6 mAb was directly conjugated to FITC. For intracellular detection of cytokine production, total blood lymphocytes were stimulated with 50 ng/mL phorbol myristate acetate (PMA; Sigma) and 2 g/mL ionomycin (Sigma) for 4 h. During the last 3 h, 10 g/mL brefeldin A (Sigma) was added. The cells were stained with APC–anti-IFN-γ (Clone B27; Biolegend) or PECy7–anti-IL-17 (clone eBio64DEC17) using the FoxP3 staining kit (eBioscience). For the detection of FoxP3, unstimulated lymphocytes were stained with APC–anti-FoxP3 (clone 259D; Biolegend) using the FoxP3 staining kit (eBioscience) The cells were analyzed on LSRII flow cytometer (BD Biosciences).

### Surface Plasmon Resonance

The SPR experiments were performed at 25°C using Bio-Rad’s ProteOn XPR36 array biosensor. A standard amine-coupling protocol was employed to immobilize Protein G’ (Sigma-Aldrich) on a GLC chip surface in 1× HBS-P buffer (10 mM HEPES, 150 mM NaCl, 0.05% (v/v) Tween 20) at a constant flow rate of 30 μl/min. A single injection of hR6; hX3 or stabilized BsAb at (5 μg/ml, 100 μl/min for 30 sec) in the vertical orientation resulted in a consistent capture of approximately 1,500 RU of proteins with no significant dissociation (drift) from the Protein G’ surface. The N-terminal peptides corresponding to the first 24 AA of hCCR6 (hCCR6 peptide) and hCXCR3 (hCXCR3 peptide) (Mimotope) were used as analyte. The peptides hCCR6 and hCXCR3 as well as an equimolar mixture of hCXCR3/hCCR6 peptides were injected simultaneously in the horizontal direction (1 μM final concentration). All SPR binding data were processed using the Scrubber-Pro software package (www.biologic.com.au).

### Chemotaxis assay

L1.2 cells stably transfected with human CXCR3 (L1.2 hCXCR3) and human CCR6 (L1.2 hCCR6) were maintained in suspension in RPMI 1640 containing 10% heat-inactivated FCS, 2% L-glutamine and 100 U ml^–1^ penicillin. For the assay, the cells were washed once in PBS and resuspended at a concentration of 10^6^ cells ml^–1^ in assay buffer (RPMI 1640, 1% endotoxin-free BSA, 100 U ml^–1^ penicillin, and 100 μg ml^–1^ streptomycin). Test antibodies (hR6: hX3 or BsAb at 1ug/ml) were preincubated with 100 μl of cells (1 × 10^5^ cells) for 30 min and placed into the upper chamber of a Transwell plate with 5-μm pores (Corning Life Sciences). In the lower chambers, 5 nM of human IP-10 and 100 nM of human CCL20 (Peprotech) (30 min, 37°C) were placed and the assay was incubated at 37°C for 4 h (5% CO_2_). Live cells migrating to the lower chamber were counted using a LSRII flow cytometer (BD Biosciences).

### Antibody-dependent cell-mediated cytotoxicity assay

The capacity of hX3 mAb; hR6 mAb and BsAb to induce ADCC was evaluated by flow cytometry as described previously [28]. Briefly, hCXCR3 and hCCR6 L1.2 expressing cells were labelled with membrane dye PKH26, to allow discrimination when incubated with effector cells and antibodies. Labelled target cells were washed three times with culture medium and resuspended in culture medium at a concentration of 1 × 10^6^ ml^–1^. Labelled target cells were dispensed in round-bottomed 96-well plates (1 × 10^5^ in 100 μl/well) and preincubated with 5 μg; 1 μg; 0.5 μg or 0.1 μg ml^–1^ of hX3; hR6; BsAb or isotype control (humanized anti-hCXCR7 antibody) at 37°C for 30 minutes. PBMCs were prepared from heparinized blood (obtained from healthy individuals) by the centrifugation of a Ficoll gradient. Human natural killer (NK) cells were next isolated from the PBMCs using MACS CD56 microbeads and MACS positive selection columns (Miltenyi Biotec) in accordance with the manufacturer's protocol.

Thereafter, 2 × 10^5^ purified human NK cells (effector cells), were incubated with the target cells/antibody mixture (E:T ratios = 4:1) at 37°C for 3 hours in the presence of 10% heat-inactivated FCS. Treated cells were washed 3 times and resuspended in 200 μl of PBS. Before analysis on a LSRII flow cytometer, TO-PRO 3 iodide was added to detect cell death. As a counting standard, 20 μl/well CountBright Absolute Counting Beads were added to determine cell concentration of specific cell subsets. Samples were acquired on an LSR II flow cytometer (BD Biosciences).

## Results

### Humanization of the murine X3 and R6 murine antibodies

Humanization is a critical step to develop therapeutic antibodies. Here, we used a method previously described [24], and grafted the murine CDRs of X3 and R6 antibodies onto human germline framework sequences. First, the VH and VL sequences were compared with the functional human germline repertoires using IMGT/V-QUEST and IMGT/Junctions analysis tools. The closest human germline sequences were then used as acceptor sequences for grafting the murine CDRs.

**Fig 1:**
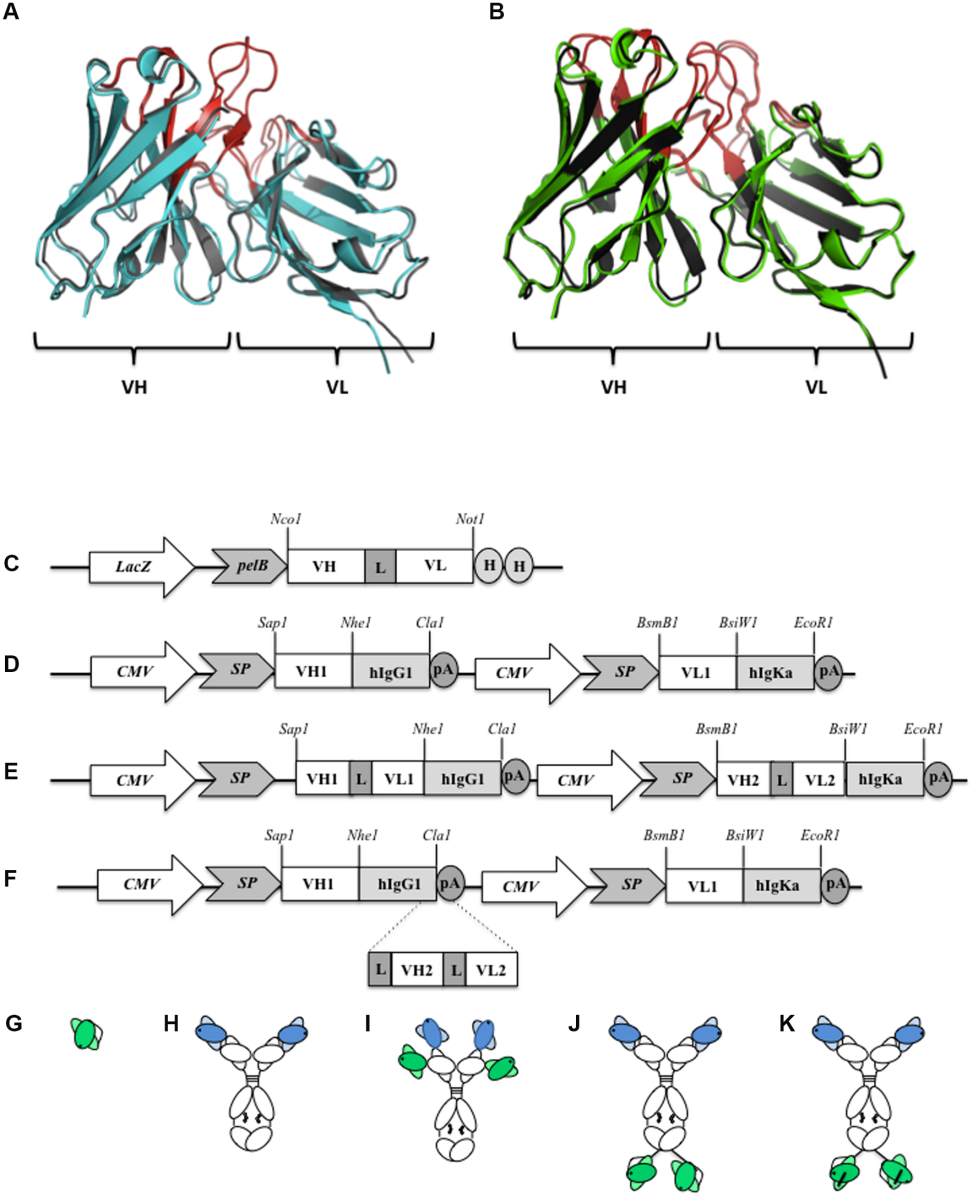
Antibody formats used in this study. Ribbon diagram representation of mX3 and mR6 Fv structure (black ribbons) superimposed with hX3 (A) (Blue ribbon) and hR6 (B) (green ribbon). The CDRs are shown in red. Schematic illustration of the vectors used for (C) scFv; (D) humanized IgG1; (E) Bispecific (scFv)_4_ IgG-like and (F) Bispecific (scFv)_2_ IgG-like expression. Abbreviations: LacZ, promoter of the bacterial Lac operon; pelB and SP, signal sequence for the secretion of the recombinant antibodies; L, (G_4_S)_3_ peptide linker; H, 6-HIS Tag; CMV, human cytomegalovirus promoter; pA, polyadenylation site. Schematic representation of the recombinant antibody formats used in this study. (G) humanized scFv; (H) humanized IgG1; (I) humanized (scFv)_4_ IgG-like bispecific antibody; (J) non stabilized humanized (scFv)_2_ IgG-like bispecific antibody and (K) stabilized humanized (scFv)_2_ IgG-like bispecific antibody.

In a second step, we back-mutated the framework residues in the “Vernier zone” that were different between the human germline and the original mouse sequences. The murine and humanized Fv domains were modelled and superimposed (Fig 1A and 1B). The final humanized VH and VL sequences were synthesized and cloned into various vector for expression in bacteria and mammalian cells.

### Design of a humanized BsAb targeting both CXCR3 and CCR6

Our aim was to design a fully humanized IgG like bispecific antibody able to target both human Th1 (through CXCR3) and human Th17 cells (through CCR6) (Fig 1A and 1B). We used different vectors to express various formats of antibodies i.e. humanized scFv; humanized IgG1 and the humanized bispecific IgG-scFv (Fig 1C–1F). The previously described pGC plasmid [25] was used to express the humanized scFv (Fig 1G) in bacteria (periplasmic expression). Our “in-house” pJacq vector was used to express a fully humanized IgG (Fig 1H) and was modified in order to express (i) Bs(scFv)4 –IgG (Fig 1I) and (ii) a IgG-like Bispecific antibody (Fig 1J and 1K). The humanized scFv and IgG1 were expressed for comparison purposes in the subsequent biochemical assays.

The first bispecific antibody we designed was expressed (Bs(scFv)4 –IgG), but not functional. Indeed, a critical step in the expression and functionality of bispecific mAb carrying scFvs is the stability of the later. Therefore, we decided to express individually the humanized R6 and X3 scFv in bacteria in order to monitor their expression. Only the hX3 scFv showed reactivity. We then designed a new bispecific construct based on the model first described by Coloma and Morrison [29]. In this model, the humanized hR6 IgG1 was used as the parent module and the hX3 scFv was fused to the C-terminal region of the heavy chain by a small (G_4_S)_3_ peptide linker. To further increase the stability of the hX3 scFv, we also designed another construct in which we introduced a disulphide bond between the VH and VL domains (VH44-VL100 disulfide bond) by site directed mutagenesis (Fig 1K).

### Expression and purification of antibodies

**Fig 2:**
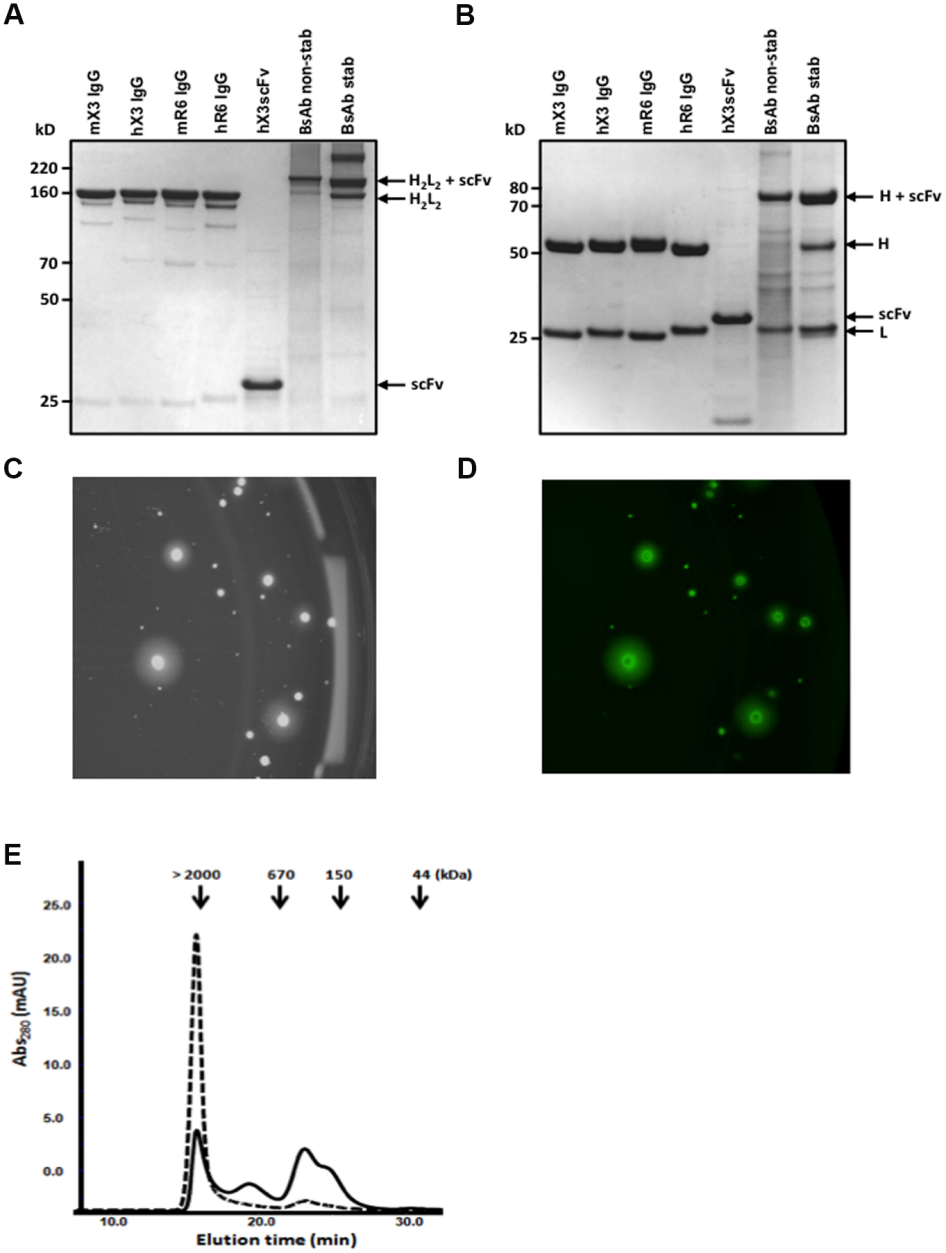
Purification of mAbs and BsAb. SDS-PAGE analysis of the various affinity purified antibodies under non-reducing conditions (A) and reducing conditions (B). The molecular weight (kDa) are shown on the left. Screening of monoclonal CHO-DG44 cells expressing the BsAb with ClonePix FL. The transfected cells were plated in a semi-solid media and visualized under bright light (C) and fluorescence (D). Size Exclusion Chromatography profile of non stabilized (dashed line) and stabilized (black line) BsAb. Arrows indicate the molecular weight of the standards.

The hX3 VH and VL genes were assembled as an scFv (VH-VL orientation) in the pGC expression vector. The construct was expressed as protein in the periplasmic space of XL-1 blue E.Coli cells and contained a C-terminal 6-HIS tag for affinity purification and detection. The SDS-PAGE shows a single band at the expected size (~30 kDa) under both non-reducing (Fig 2A) and reducing conditions (Fig 2B) for the scFv, purified by immobilized metal-affinity chromatography (IMAC).

For the humanized hX3 and hR6 IgG expression, the VH and VL gene were sequentially cloned into the pJacq mammalian expression vector and the resulting plasmids were linearized and stably transfected in CHO-DG44 cells by electroporation. Two weeks after transfection, the cells were replated in a selective semi-solid media and screened individually for high producing subclones using a Clonepix FL. The monoclonal CHO cells were selected based on expression level of antibodies detected by the fluorescent ‘halo’ around the individual colonies (Fig 2C and 2D). Selected clones were produced and SDS-PAGE analysis of the purified humanized antibodies showed a similar profile to the parental murine antibodies with a main characteristic band at ~150 kDa (non-denaturing conditions) and two bands at ~25 kDa (light chains) and ~ 50 kDa (heavy chains) under denaturing conditions (Fig 2A and 2B).

For expression of the BsAb, (G_4_S)_3_-hX3 scFv cassettes (non-stabilized and stabilized) were inserted in the hR6 IgG pJaq expressing vector. Monoclonal CHO-expressing cells were selected and purified as describe above. Both the non-stabilized and stabilized BsAb showed an apparent molecular weight of ~200 kDa upon SDS-PAGE analysis under non-reducing conditions (Fig 2A). Under reducing conditions (Fig 2B), both BsAb gave rise to two main bands at ~ 75 kDa (Heavy Chain + scFv) and ~ 25 kDa (Light Chain). Interestingly, the non-stabilized BsAb showed numerous other bands and a “smeary” profile compared to the stabilized BsAb, indicating proteolysis. Those observations correlated with the analytical Size Exclusion Chromatography (SEC) profile (Fig 2E) following protein A purification. Indeed, the non-stabilized BsAb appears to be mainly constituted of aggregates. The stabilization of the BsAb reduces the amount of aggregated materials and increases the quantity of correctly folded BsAb. The BsAb fraction (Fig 2E; grey rectangle) was isolated and used in further assays *in vitro*.

### Surface Plasmon Resonance binding assay demonstrates reactivity of CXCR3.CCR6 BsAb

**Fig 3:**
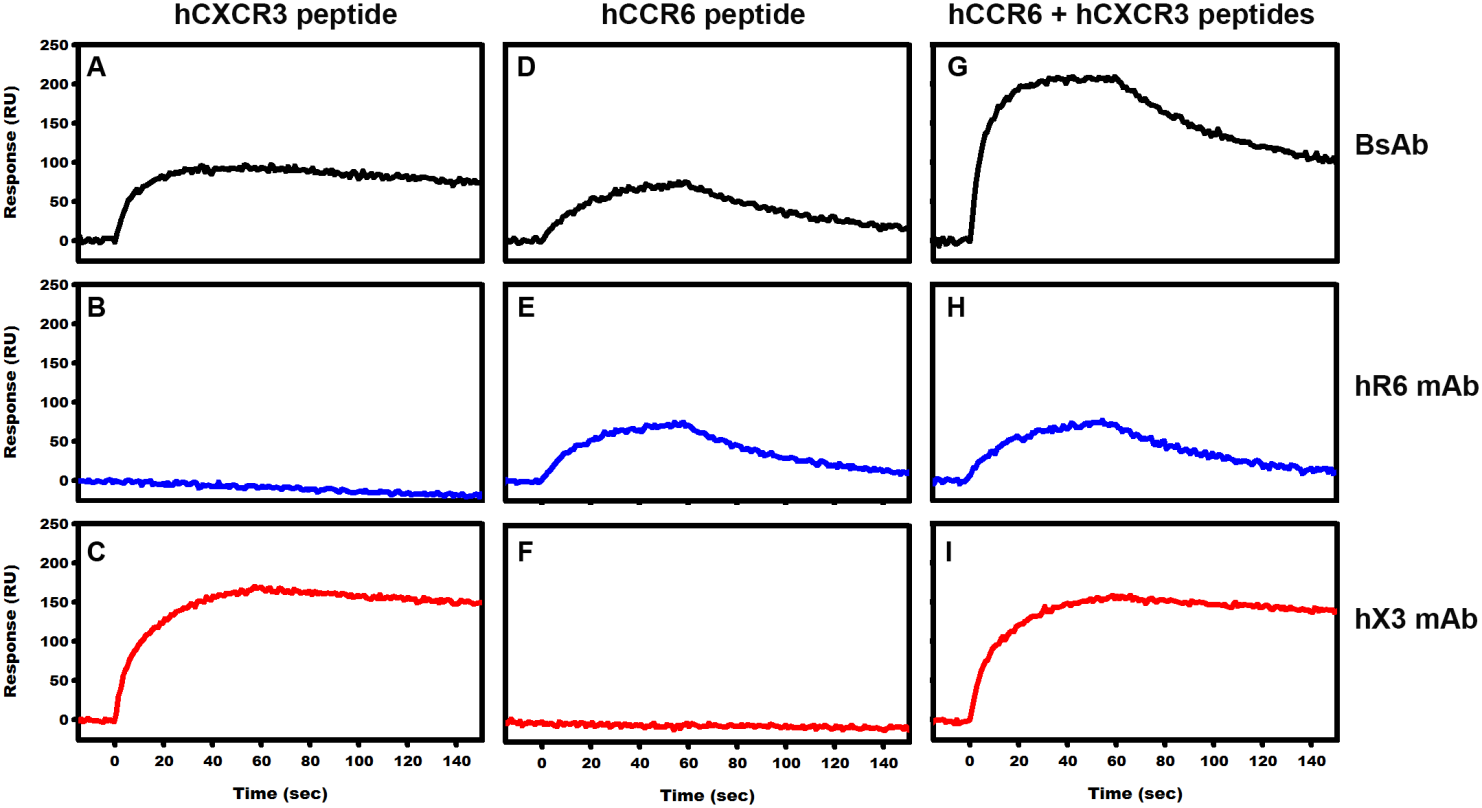
Surface Plasmon Resonance analysis. SPR experiment showing binding of hCXCR3 and hCCR6 peptides to bispecific stabilized antibody and to control parental humanized IgG’s. GLM chip containing immobilized Protein G’ docked in ProteOn XPR3 instrument was utilized for this experiment. BsAb, hX3 and hR6 IgG1 were captured onto protein G’ chip surface via their Fc regions. hCXCR3 peptide (Panels A, B, C), hCCR6 peptide (Panels D, E, F) and a mixture of hCCR6 and hCXCR3 peptides (G,H,I) were injected over immobilized bispecific Ab (top panels, black curves), hR6 IgG1 (middle panels, blue curves) and hX3 (bottom panels, red curves).

The ability of the BsAb to bind both antigens of CXCR3 and CCR6 simultaneously was next demonstrated by SPR. The BsAb (Fig 3, top panel), hR6 IgG1 (middle panel) and hX3 IgG1 (bottom panel) were captured via their Fc region on a Protein G immobilized chip on three different channels. The analytes (hCXCR3; hCCR6 or hCXCR3 + hCCR6 peptides) were next injected simultaneously in opposite directions over the immobilized recombinant proteins. As seen in Fig 3, only the captured BsAb (Fig 3A) and hX3 IgG1 (Fig 3C) bound to the hCXCR3 peptide, while hR6 IgG1 did not (Fig 3B). Similarly, when hCCR6 peptide was used as analyte, a response was observed for captured BsAb (Fig 3D) and hR6 (Fig 3E), but not for hX3 (Fig 3F). When a mixture of hCXCR3 and hCCR6 peptides was used, hLJR6 (Fig 3H) and hX3 (Fig 3I) showed a binding curve identical to the ones observe when only their respective target was injected alone, indicating that they reacted with only one peptide in the mixture. Importantly, in a similar assay configuration, the captured BsAb showed a binding response corresponding to the sum of the ones observed, when the single peptides where injected individually (Fig 3G). Thereby, we confirmed that the BsAb effectively targets simultaneously both antigens.

### BsAb stains both CXCR3 and CCR6 expressing cells by flow cytometry

**Fig 4:**
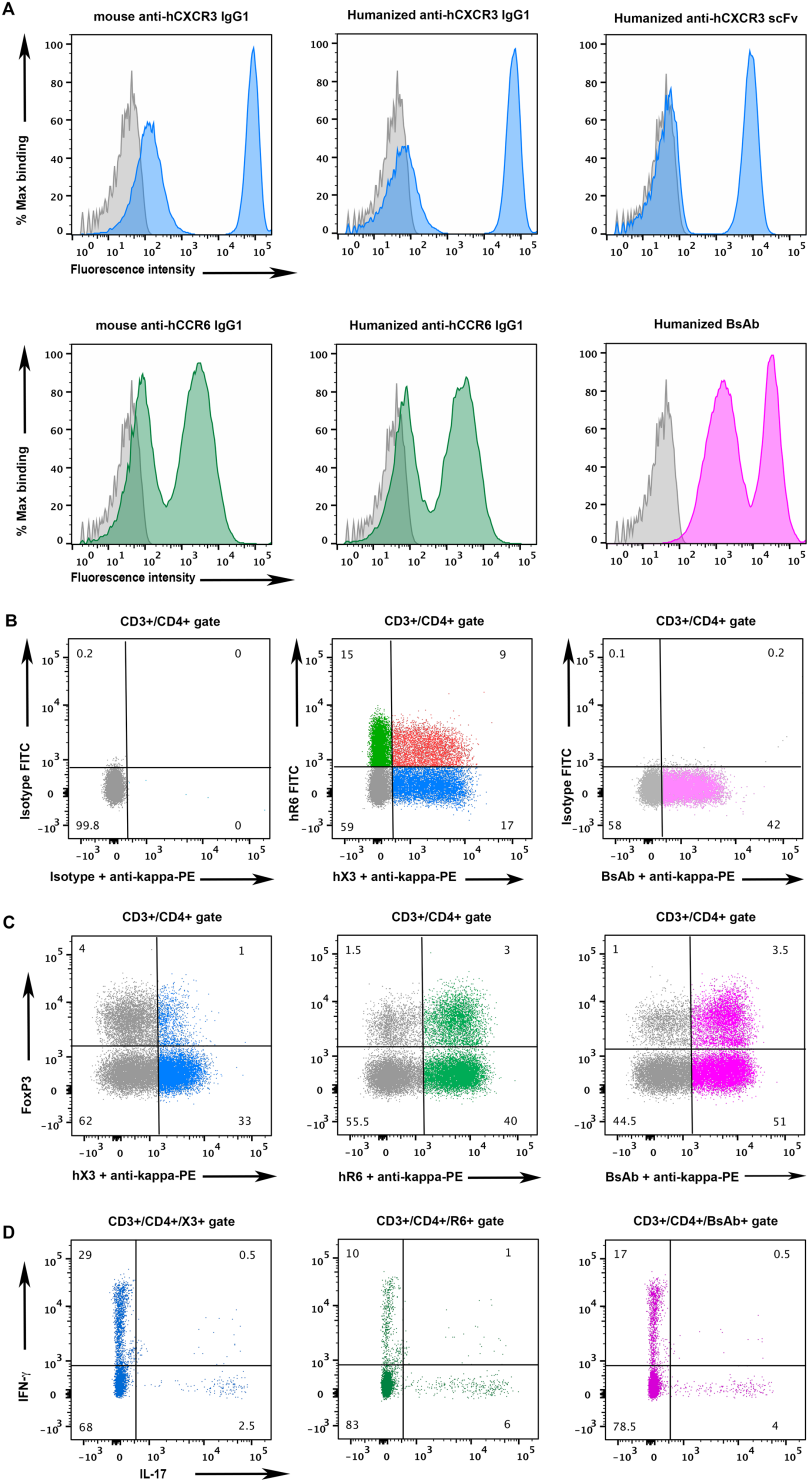
Flow cytometric analysis. (A) Reactivity of mAb and BsAb on hCXCR3 and hCCR6 L1.2 expressing cells. The hCXCR3 and hCCR6 L1.2 expressing cells were mixed and incubated with an isotype control antibody (grey line) or mX3 mAb IgG1 (blue line; top left panel); hX3 IgG1 (blue line; top middle panel); hX3 scFv (blue line; top right panel); mR6 IgG1 (green line; bottom left panel); hR6 IgG1 (green line; bottom middle panel) or BsAb (purple line; bottom right panel). The murine IgG1 were stained with an anti-mouse igG Fc specific PE-conjugated; the humanized scFv was stained with an anti-6HIS antibody followed by an anti-mouse igG Fc specific PE-conjugated antibody; the humanized IgG1 and BsAb were stained with an anti-human kappa PE conjugated antibody. (B) Reactivity of mAb and BsAb on purified human lymphocytes. Human lymphocytes were isolated from blood on a ficoll gradient and stained with an anti-human CD3-BV650 and an anti-human CD4 Pacific Blue conjugated antibody. The lymphocytes in the CD3+/CD4+ gate were stained with isotype controls (left panels); FITC conjugated hR6 mAb and hX3 (detected with anti-human Kappa PE-conjugated antibody) (middle panel) or BsAb (right panel) followed by an anti-human Kappa PE-conjugated antibody. (C) Intracellular staining for FoxP3 protein in lymphocytes isolated from human blood. Activated human lymphocytes were gated on CD3+/CD4+/hX3+ (left panel); CD3+/CD4+/hR6+ (left panel); CD3+/CD4+/BsAb+ (left panel) and stained intracellularly for FoxP3. (D) Intracellular staining for IFN-γ and IL-17A proteins in PMA/ionomycin-stimulated lymphocytes isolated from human blood. Activated human lymphocytes were gated on CD3+/CD4+/hX3+ (left panel); CD3+/CD4+/hR6+ (left panel); CD3+/CD4+/BsAb+ (left panel) and stained intracellularly for IFN-γ and IL-17A.

To investigate whether our BsAb was able to bind simultaneously to hCXCR3 and hCCR6, a flow cytometric assay was used. The BsAb dual specificity was monitored using L1.2 cells expressing hCCR6 or hCXCR3. In this assay, L1.2 hCXCR3 and L1.2 hCCR6 cells were mixed in equal proportions and stained with our scFv stabilized BsAb. As controls, we included the mouse parental anti-hCXCR3 and anti-hCCR6 mAb, as well as the corresponding humanized IgG1 mAbs and the humanized hX3 scFv. For the humanized mAb and the BsAb, the binding was revealed with an-anti-human kappa light chain PE-conjugated antibody in order to confirm the correct folding of the molecules. For the mouse IgGs, we used an anti-mouse Fc PE-conjugated antibody. For the scFv we used an anti-6-HIS antibody in combination with an anti-mouse Fc PE conjugated antibody, since the anti-kappa antibody is unable to bind to the VL domain alone. The results confirmed that the monovalent (scfv) and divalent antibodies (mouse and humanized IgGs) were able to recognize only one cell population (Fig 4A). On the other hand, when the cells were incubated with the tetravalent BsAb, a clear shift of both cell populations was observed and the fluorescence intensity was similar to the one observed with the murine and humanized IgGs. These results demonstrate that our BsAb bound both receptors, as previously shown by SPR.

We next confirmed the dual binding capacity of our BsAb to the native receptors CXCR3 and CCR6, expressed by human Th1 and Th17 subsets, respectively (Fig 4B). Human blood lymphocytes from patients aged over 50 were purified on a Ficoll gradient and stained with anti-CD3, anti-CD4 and hX3 + hR6 or BsAb. As shown in Fig 4B, ~ 28% of CD3+/CD4+ lymphocytes express CXCR3 while ~ 24% of them express CCR6 and ~ 9% co-expressed both receptors. Interestingly, when the CD3+/CD4+ cells were stained with the BsAb, 42% of the cells were positive, indicating that all three CD4+/CXCR3-/CCR6+; CD4+/CXCR3+/CCR6- and CD4+/CXCR3+/CCR6+ populations were stained. We next investigate the FoxP3 expression of CD3+/CD4+/X3+; CD3+/CD4+/R6+ and CD3+/CD4+/BsAb+ populations (Fig 4C). We noticed that BsAb mAb could stain a large proportion of Tregs (~ 3.5% of the CD3+/CD4+ subset) compare to X3 (~ 1%) and R6 (~ 3%) mAbs.

To evaluated the intracellular expression of the cytokines IL-17 and IFN-γ, by CD4+/hR3+; CD4+/hR6+ and CD4+/BsAb+ subsets, we analyzed freshly isolated PBMCs and activated them with PMA and Ionomycin (Fig 4D). T cells were permeabilized, and their cytokine profile was studied by flow cytometry and multicolor intracellular staining. As expected, the hR3+ subset was characterized by a large proportion of IFN-γ producing cells (Th1 cells; ~ 30%) whereas the hR6+ contains both IFN-γ (~ 10%) and IL-17 producing cells (Th17; ~ 6%). Interestingly, the BsAb+ subset was characterized by a higher proportion of IFN-γ producing cells than the hR6+ subset (17% vs 10%) and a higher proportion of IL-17+ cells than the hR3 subset (4% vs 2.5%). However, in all subsets, the amount of IFN- γ+/IL-17+ cells (Th17.1) was low (0.5–1%) indicating that in healthy patients, this population is rare.

### BsAb inhibits CXCR3- and CCR6-mediated cell migration

**Fig 5:**
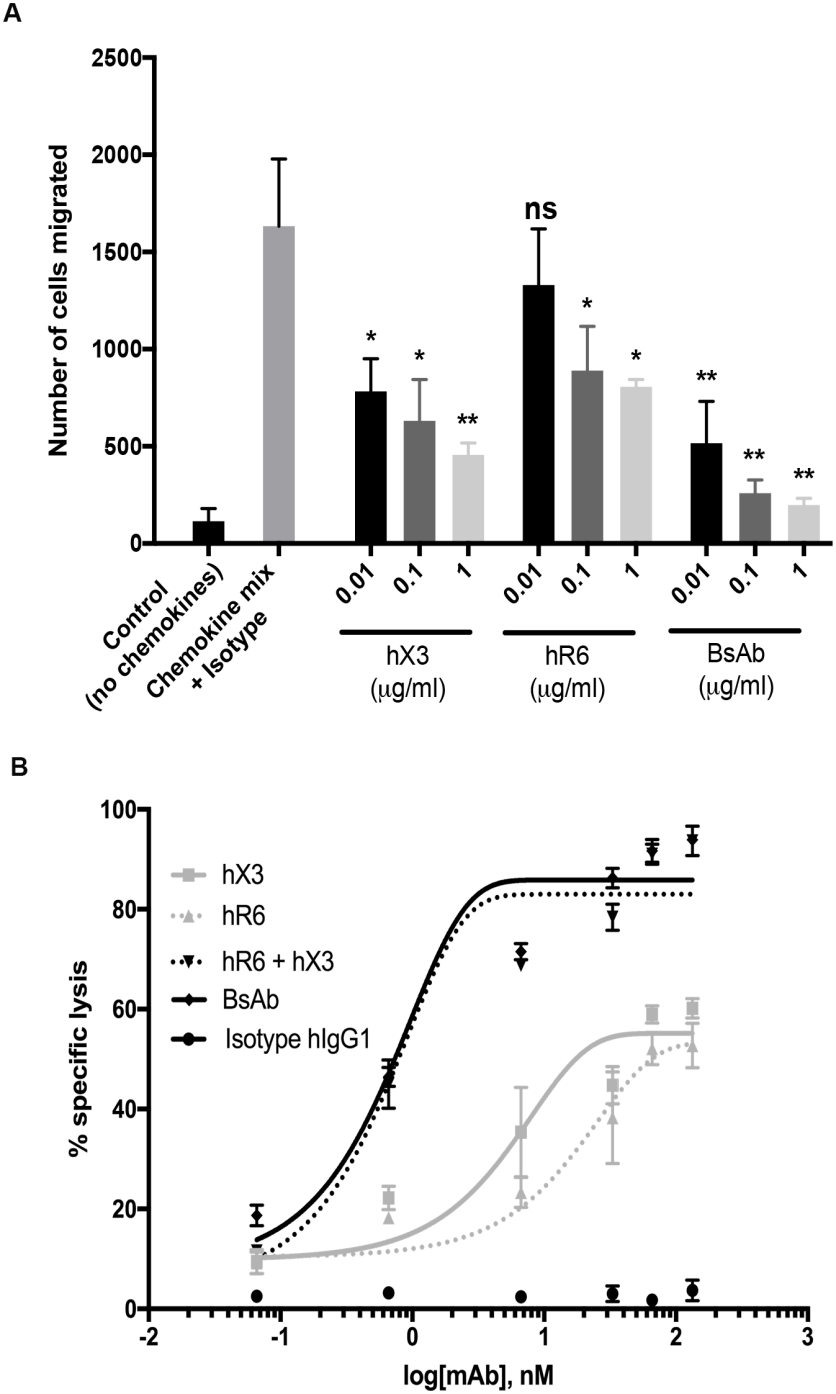
In vitro characterization of mAbs functions. Chemotaxis inhibition by hX3; hR6 and BsAb. (A) The hCCR6 and hCXCR3 L1.2 cells were mixed; preincubated with an Isotype control and allowed to migrated towards a combination of hIP-10 and hCCL20 chemokines in the lower chamber. The hCCR6 and hCXCR3 L1.2 cells chemotaxis to hCCL20 and hIP-10 was carried out in the presence of increasing concentrations of purified R6; X3 and BsAb. Statistical analyses were performed using a t-test comparison between Isotype and antibodies groups (* p<0.05, ** p<0.01). (B) Cytotoxicity of activated human NK cells (effector) against a mixture of hCCR6 and hCXCR3 L1.2 cells (target) by R6; X3; R3 + X3 and BsAb *in vitro*.

To examine the functional capability of our BsAb to inhibit the simultaneous migration of hCXCR3 and hCCR6 L1.2 expressing cells *in vitro*, a chemotaxis assay was performed (Fig 5A and 5B). The BsAb was incubated at various concentrations (1; 0.1 and 0.01 μg/ml) with a mixture of hCXCR3 and hCCR6 L1.2 expressing cells in the upper chamber of a chemotaxis plate. Cells were then allowed to migrate towards the human IP-10 and human MIP-3α localized in the lower chamber. As controls, hX3 mAb, hR6 mAb and a human IgG1 isotype (1 μg/ml) were used in the assay.

As shown in Fig 5A, hX3 and hR6 exhibit an inhibitory effect compared to the isotype control. More importantly when the cells were incubated with the bispecific mAb, we observed an important reduction of the cell migration. Thus, our BsAb binds to both CXCR3 and CCR6 and inhibit cell chemotaxis.

### BsAb is cytotoxic for both CXCR3 and CCR6 expressing cells

We studied the specific cytotoxic activity of our BsAb using a flow cytometric based assay as described previously [28]. We used purified Natural Killer (NK) cells as effector cells and a mixture of PKH-26 labelled L1.2 hCXCR3 and L1.2 hCCR6 cells as targets. In our hands, the best effector/target ratio was 4:1. The dead cells were labelled with TOPRO-3 dye and therefore the specific dead target cells were monitored in the PKH-26+/TOPRO-3+ gate. As shown in Fig 5B, a dose-dependent specific cell cytoxicity was observed with all antibodies except the human IgG1 isotype control. When we used a high concentration of humanized hX3 or hR6, the proportion of dead target cells did not exceed 50%, confirming that hX3 and hR6 were not able to mediate antibody dependant cell cytotoxicity (ADCC) towards hCXCR3 and hCCR6 L1.2 expressing cells simultaneously, as expected. In contrast, our BsAb showed pronounced cytotoxic activity (~ 80–90% specific cell death) at concentration of 1 μg/ml. Taken together we conclude that (i) the addition of the scFv to the C-terminal region did not impair binding of the BsAb to the Fc receptor and, (ii) our BsAb was able to specifically induce ADCC toward hCXCR3 and hCCR6 L1.2 target cells in the presence of human NK effector cells.

## Discussion

Due to the redundancy of chemokine receptors, their specific targeting with monoclonal antibodies can be more beneficial than targeting their respective ligands. However, in many pathological conditions, multiple chemokines receptors are involved in the migration of pro-inflammatory leukocytes. This is the case of CXCR3 and CCR6, which are associated with the recruitment of pathological Th1; Th17 and Th17.1 in various inflammatory and autoimmune disorders. Our aim was to generate a bispecific antibody with the ability to simultaneously inhibit and/or deplete immune cells that express both receptors, which may represent an effective therapeutic approach in aforementioned disorders.

Bispecific antibodies have been particularly successful in oncology, for instance to assist the recruitment of cytotoxic T cells towards the site of the tumor [30]. Recently, their therapeutic use has been extended to inflammatory and autoimmune diseases including asthma [31, 32], Alzheimer’s disease [33], inflammatory arthritis [34] or psoriasis [35], at least in animal models.

Three main strategies have been established to produce bispecific antibodies: (i) hybrid hybridomas, (ii) chemical conjugation and (iii) antibody engineering. However, the hybrid hybridomas technique leads to the formation of numerous misassembled antibody species and the chemical conjugation approach is frequently associated with low production yields. On the other hand, antibody engineering has allowed the development of more than 45 different bispecific formats based on the modular architecture of immunoglobulins [19]. Small bispecific antibodies such as diabodies can be generated by combining the FV regions of two antibodies. However, those formats generally lack the Fc region resulting in poor pharmacokinetic properties and limited effector functions. To circumvent this problem, other larger formats called IgG-like bispecific antibodies have been described. They are usually produced through the fusion of an scFv to a mAb resulting in a tetravalent molecule with increase avidity. IgG-like bispecific antibodies show similar pharmacokinetic behavior and effector functions compared to regular mAbs.

In the present study, our aim was to design and characterize a fully humanized bispecific antibody, capable of simultaneously recognizing hCXCR3 and hCCR6. In order to decrease the potential immunogenicity of our construct, we first humanized the parental murine VH and VL gene using a previously described approach and expressed them as full length human IgG1. The humanized mAbs both showed a specific staining by flow cytometry similar to the mouse mAbs.

In our study, we wanted to keep the effector functions fully intact and therefore chose an “IgG like BsAb” format, comprising a human Fc region, over a small diabody format. Given that our first attempt to express a Bs(scFv)4 [36] was unsuccessful, we decided to use the “Morisson” bispecific format [29], in which hR6 IgG1 is used as the parent module with the hX3 scFv fused to the C terminal region of the constant heavy chain. This tetravalent structure was expressed and functional, however, SEC profile revealed a high proportion of aggregated products. Based on previous studies, we chose to stabilize the scFv by introducing an extra disulfide bridge between the VH and VL domains [37]. This newly designed construct showed better biophysical characteristics despite the presence of aggregation products and further optimization steps will be necessary. Indeed, Schanzer et al. [38] have shown that by increasing the linker length in the scFv moiety, it is possible to further decrease the amount of aggregation. However, we explicitly confirmed that our purified BsAb is able to bind simultaneously to CXCR3 and CCR6, as demonstrated by flow cytometry and SPR assays.

The mechanisms of action of therapeutic mAb can be divided into two main categories: (i) blocking the action of the target upon binding or (ii) depleting the cells expressing the target via the Fc effector functions. Accordingly, we analyzed the capability of our BsAb to (i) inhibit the migration of CXCR3 and CCR6 expressing cells in a chemotaxis assay and (ii) to specifically induce ADCC. We demonstrated that the BsAb showed remarkable efficacy in both assays. Importantly, those functions can be refined by using well-described Fc-engineering technology.

Altogether, the results demonstrate the feasibility of constructing a BsAb that can effectively and simultaneously target CXCR3 and CCR6, two important markers for subets of effector T cells. These two receptors are strongly associated with pro-inflammatory Th1; Th17 T-cell subsets. It is conceivable that approaches using BsAbs, as outlined here, will expand the utility of mAb based drugs, and allow circumvention of chemokine/receptor redundancy for the treatment of various inflammatory and autoimmune disorders.
